# Anticancer Activity of Astaxanthin-Incorporated Chitosan Nanoparticles

**DOI:** 10.3390/molecules29020529

**Published:** 2024-01-21

**Authors:** Eun Ju Hwang, Young-IL Jeong, Kyong-Je Lee, Young-Bob Yu, Seung-Ho Ohk, Sook-Young Lee

**Affiliations:** 1 Marine Bio Research Center, Chosun University, Wando 59146, Jeonnam, Republic of Korea; ejlife0827@naver.com; 2 Research Institute of Convergence of Biomedical Sciences, Pusan National University Yangsan Hospital, Yangsan 50612, Gyeongnam, Republic of Korea; nanomed@naver.com; 3 Department of Prosthodontics, Chosun University Dental Hospital, Gwangju 61452, Republic of Korea; lkj1998@chosun.ac.kr; 4 Department of Paramedicine, Nambu University, Gwangju 62271, Republic of Korea; ybyu@nambu.ac.kr; 5 Department of Oral Microbiology, Chonnam National University School of Dentistry, Gwangju 61452, Republic of Korea

**Keywords:** astaxanthin, ROS scavenging, anticancer, chitosan, nanoparticle

## Abstract

Astaxanthin (AST)-encapsulated nanoparticles were fabricated using glycol chitosan (Chito) through electrostatic interaction (abbreviated as ChitoAST) to solve the aqueous solubility of astaxanthin and improve its biological activity. AST was dissolved in organic solvents and then mixed with chitosan solution, followed by a dialysis procedure. All formulations of ChitoAST nanoparticles showed small diameters (less than 400 nm) with monomodal distributions. Analysis with Fourier transform infrared (FT-IR) spectroscopy confirmed the specific peaks of AST and Chito. Furthermore, ChitoAST nanoparticles were formed through electrostatic interactions between Chito and AST. In addition, ChitoAST nanoparticles showed superior antioxidant activity, as good as AST itself; the half maximal radical scavenging concentrations (RC_50_) of AST and ChitoAST nanoparticles were 11.8 and 29.3 µg/mL, respectively. In vitro, AST and ChitoAST nanoparticles at 10 and 20 µg/mL properly inhibited the production of intracellular reactive oxygen species (ROSs), nitric oxide (NO), and inducible nitric oxide synthase (iNOS). ChitoAST nanoparticles had no significant cytotoxicity against RAW264.7 cells or B16F10 melanoma cells, whereas AST and ChitoAST nanoparticles inhibited the growth of cancer cells. Furthermore, AST itself and ChitoAST nanoparticles (20 µg/mL) efficiently inhibited the migration of cancer cells in a wound healing assay. An in vivo study using mice and a pulmonary metastasis model showed that ChitoAST nanoparticles were efficiently delivered to a lung with B16F10 cell metastasis; i.e., fluorescence intensity in the lung was significantly higher than in other organs. We suggest that ChitoAST nanoparticles are promising candidates for antioxidative and anticancer therapies of B16F10 cells.

## 1. Introduction

Astaxanthin (AST), which is a blood-red pigment, is a natural compound produced in marine microalgae [1,2]. Due to its biological activity, AST has been used in various industries, including aquaculture, food, cosmetics, nutraceuticals, and pharmaceuticals [2,3,4]. For example, AST is known as one of the most important pigments of salmon, trout, and shrimp meat because the color of the fish affects consumers’ preferences around the world [3]. In particular, its biological activities, such as pigmentation, ultraviolet (UV) light protection, immune response, antioxidative effects, reproductive capacity, and stress tolerance, have been spotlighted in the field of the biomedical and health food additive industries [5]. The biological activity of AST is basically related to a hydroxyl group and a keto group on each end of its chemical structure; its peculiar features promote human health [6,7]. In particular, unique chemical structures enable it to scavenge reactive species of oxygen or nitrogen, i.e., polar end groups and double bonds of the middle segment are contributed to quench free radicals and remove high-energy electrons, respectively [7,8]. Due to its intrinsic features, AST has superior antioxidant activity compared to other carotenoids [6,7,8,9]. Its unique features as an antioxidant provide various biological activities in the biomedical field [9]. For example, AST has antibacterial activity against pathogens, i.e., it reduces bacterial load and gastric inflammation in *Helicobacter pylori* (*H. pylori*)-infected mice [10]. Also, it suppresses reactive oxygen species (ROS) levels and inteleukin-8 (IL-8) expression levels in cells [11]. Furthermore, AST is known to stimulate the immune function [12,13]. Yin et al. reported that astaxanthin reduces lipopolysaccharide (LPS)-induced inflammatory cytokines and then abrogates allogeneic T cell proliferation [13]. The anticancer efficacy of AST was also reported by several authors [14,15,16,17,18]. Natural AST showed inhibitory effects against the proliferation of renal clear cell carcinoma and placental growth factor expression [14]. Ramamoorthy et al. reported that AST induces cell cycle arrest, lysosomal acidification, and apoptosis of A549 cells [15]. Also, Tsuji et al. reported that glioblastoma progression in a murine orthotopic model was suppressed by oral administration of AST [16]. In spite of these advantages, some side effects, such as increased bowel movements, abdominal/stomach pain, itch, dyspepsia, muscle pain, and/or diarrhea may limit its application in humans [17,18,19]. Furthermore, low aqueous solubility (less than 2 µg/mL in pH 6.5 solution) and low stability are also problematic for practical application in humans [20,21]. To solve the disadvantages and problems of AST, various formulations based on drug delivery systems have been investigated [22,23,24]. Polyakov and Kispert reported that inclusion complexes based on polysaccharides increase aqueous solubility and photostability [22]. Slonimskiy et al. reported that AST-binding protein solubilizes AST, and that its photo/biological activity can be modulated [24].

Nanodimensional carriers have been extensively investigated to develop improved drug delivery systems [25,26,27,28]. Since nanoparticles have a small diameter, they have advantages in avoiding the reticuloendothelial system (RES), solubilizing lipophilic agents, passive/active disease targeting, and offering ease of surface modification and payload bioactive agents [25,26,27,28]. For example, chitosan nanoparticles are known to increase the aqueous solubility of retinol and improve its bioavailability [27]. Jia reported that nanoparticle formulation enhances the oral bioavailability of poorly water-soluble drugs and thus their therapeutic effectiveness [28]. Antibiotic-encapsulated poly(dl-lactide-co-glycolide) (PLGA) nanoparticles improved the antibacterial activity of ciprofloxacin in an in vivo animal model compared to free ciprofloxacin [29]. Ion complex nanoparticles between hyaluronic acid and cisplatin selectively released anticancer drugs in a tumor-enzyme sensitive manner [30]. Nanoencapsulation of AST is also one of the key features in solving these problems [31,32]. Nanoencapsulation enables the protection of AST from chemical structure damage by long-term storage and degradation by digestion [31]. Nanoliposol formulation provides increased aqueous solubility and antioxidant activities [32].

In this study, we fabricated AST-incorporated nanoparticles using glycol chitosan (GC) through electrostatic interactions. Then, their antioxidant activity and stability were evaluated in vitro using murine macrophage cells. Furthermore, the anticancer activity of AST-incorporated GC nanoparticles was evaluated with human melanoma cancer cells.

## 2. Results

### 2.1. Characterization of Astazanthin-Incorporated Chitosan (ChitoAST) Nanoparticles

**Figure 1:**
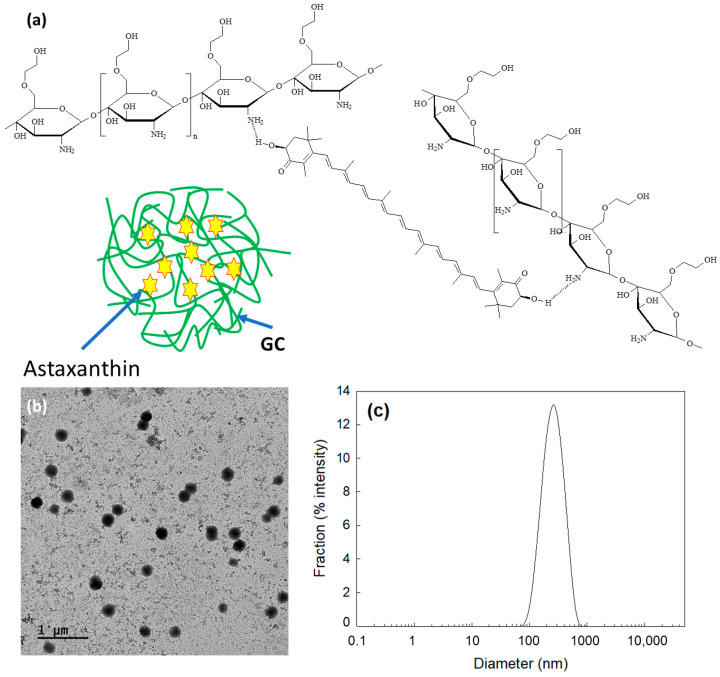
(**a**) Schematic illustrations of electrostatic interaction between hydroxyl group of AST and amine group of chitosan. (**b**) Morphological observation of ChitoAST-2 nanoparticles. (**c**) Particle size distribution of ChitoAST nanoparticles.

ChitoAST nanoparticles were prepared based on ion complex formation between AST and glycol chitosan (Chito) (Figure 1a). Their characteristics are summarized in Table 1. As shown in Table 1, ChitoAST nanoparticles have small diameters of less than 400 nm. When the feeding weight of AST was increased, the loading efficiency gradually increased. The zeta potential of the nanoparticles was changed by minor degrees according to the content of AST in the nanoparticles, as shown in Table 1. Particle size decreased according to the increase in AST content in the nanoparticles, as shown in Table 1. These results might be due to the fact that AST in the matrix of nanoparticles forms hydrophobic interactions at higher drug contents because AST is a lipophilic drug. Since the hydrophobicity of AST and AST and Chito form relatively tight complexes in the nanoparticle matrix, the size of the nanoparticles could be decreased. Otherwise, hydrophilic segments such as Chito are relatively more swellable in the aqueous solution at lower contents of AST, so this property must induce an increase in particle size, as shown in Table 1.

As shown in Figure 1b, ChitoAST nanoparticles have spherical shapes and small diameters. Furthermore, their sizes were about 100~300 nm. As shown in Figure 1c, the particle size distribution of ChitoAST nanoparticles reveals a monomodal distribution pattern, and their average sizes are less than 400 nm. These results indicate that AST and chitosan successfully formed spherical nanoparticles. 

**Figure 2:**
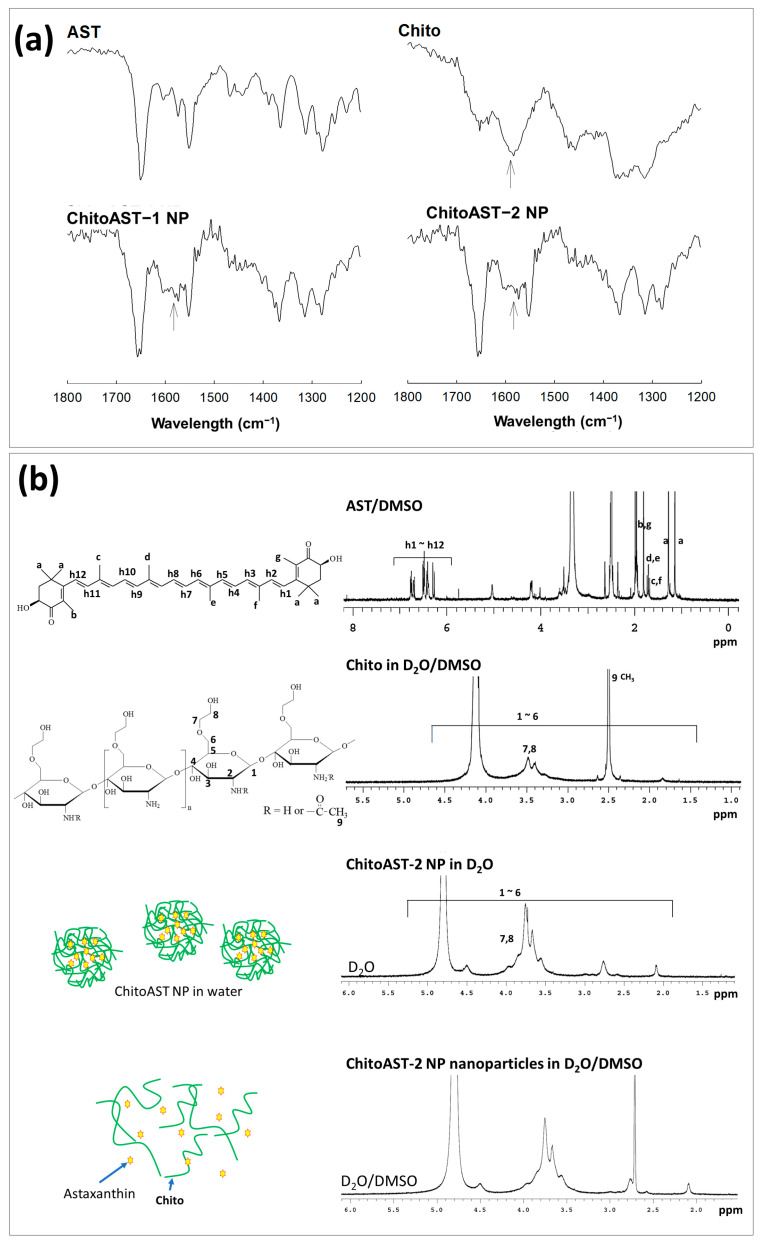
(**a**) Fourier transform infrared (FT−IR) spectra of AST, Chito, ChitoAST-1 and ChitoAST-2 nanoparticles. NP = nanoparticles. (**b**) ^1^H NMR spectra of AST (DMSO), Chito (D_2_O/DMSO), ChitoAST-2 nanoparticles (D_2_O) and ChitoAST-2 nanoparticles (D_2_O/DMSO).

Figure 2a shows the Fourier transform infrared (FT-IR) spectra of the ChitoAST nanoparticles. As shown in Figure 2a, AST and chitosan show their specific peaks between 500 cm^−1^ and 3000 cm^−1^, i.e., AST shows its specific peak at about 1700 cm^−1^ while chitosan shows its specific peak at 1590 cm^−1^. As shown in Figure 2, the single peak of chitosan at around 1590 cm^−1^ changes to a semi-doublet (arrows in Figure 2a), indicating that ChitoAST nanoparticles must have been formed through electrostatic interaction between the amine group of chitosan and the hydroxyl group of AST, as illustrated in Figure 1a. Furthermore, AST and ChitoAST nanoparticles were analyzed with ^1^H nuclear magnetic resonance (NMR) spectra (Figure 2b). AST has its specific peaks between 1.0 ppm and 7.0 ppm, while GC itself shows specific peaks at 1.5~4.5 ppm. When ChitoAST-2 nanoparticles are in aqueous solution (ChitoAST-2 NP in D_2_O), Chito peaks are primarily observed. However, specific peaks of AST were observed between 2.5~3.0 ppm in D_2_O/DMSO. These results indicated that AST is incorporated into the nanoparticle matrix.

**Figure 3:**
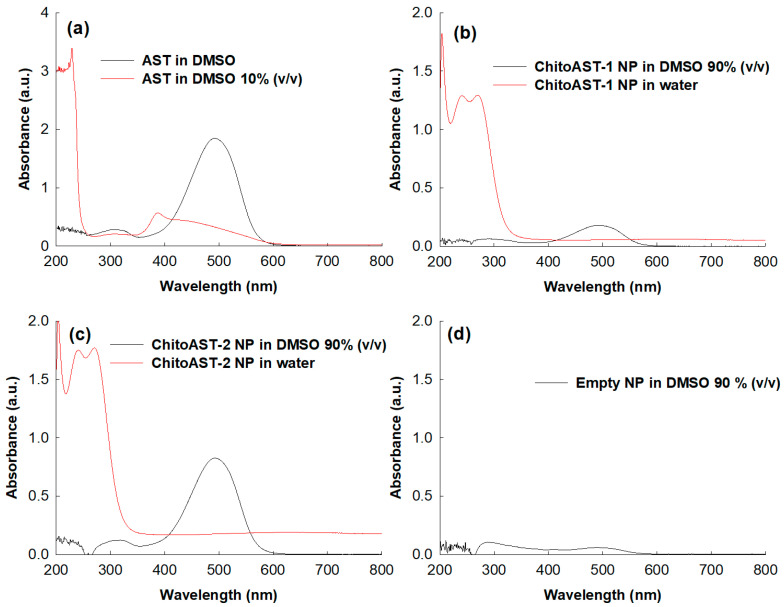
UV spectrum of AST and ChitoAST-2 nanoparticles. (**a**) AST in DMSO. AST (10 μg/mL in DMSO or DMSO/water mixtures (1/9, *v*/*v*). (**b**) ChitoAST-1 nanoparticles (ChitoAST-1 NP) in DMSO/water mixtures (9/1, *v*/*v*) or water. (**c**) ChitoAST-2 nanoparticles (ChitoAST-2 NP) in DMSO/water mixtures (9/1, *v*/*v*) or water. (**d**) Empty nanoparticles (Empty NP, Chito only) in DMSO/water mixtures (100 μg/mL in DMSO/water mixtures (9/1, *v*/*v*)).

Figure 3 shows the UV spectra of the AST and ChitoAST-2 nanoparticles. As shown in Figure 3a, AST shows intrinsic absorption peaks at UV spectra, and its maximum absorption peak was recorded at around 480~490 nm at DMSO. However, the absorption spectra of AST were quite different when DMSO concentration was 10% (*v*/*v*) and a maximum peak was observed around 380 nm. Figure 3b,c show the UV spectra of ChitoAST-1 and ChitoAST-2 nanoparticles. As shown in Figure 3b,c, ChitoAST nanoparticles show a quenched absorption spectrum in water, but they show a similar absorption spectrum in DMSO (90%, *v*/*v*) compared to AST itself, i.e., they also reveal a maximum absorption peak at around 480~490 nm. Practically, empty nanoparticles—virtually Chito itself—showed a negligible broad spectrum between 200 nm and 800 nm, as shown in Figure 3d. These results indicate that AST ChitoAST nanoparticles maintain the intrinsic absorption spectrum of AST itself during nanoparticle preparation.

**Figure 4:**
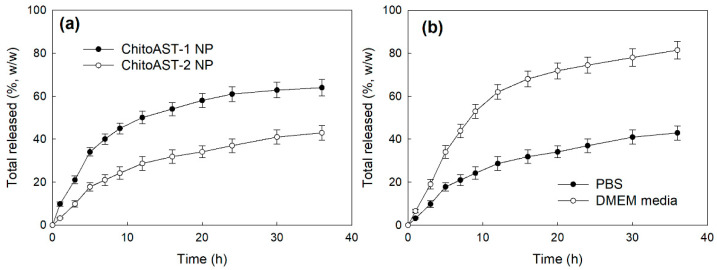
AST release from ChitoAST nanoparticles. (**a**) The effect of drug content; (**b**) The effect of media on the drug release characteristics (ChitoAST-2 nanoparticles).

Figure 4 shows the drug release from the ChitoAST nanoparticles. As shown in Figure 4a, the higher the drug content in the nanoparticles induced, the slower the drug release rate. Furthermore, burst release properties were observed at 9 h, and then AST was slowly released from the nanoparticles over 2 days. When DMEM media was used as a release medium, the drug release rate was higher than that in PBS, as shown in Figure 4b. Furthermore, burst release was observed until 9 h, and then drug release was continued over 2 days.

### 2.2. Antioxidants of ChitoAST Nanoparticles

Table 2 shows the 2,2′-azino-bis(3-ethylbenzothiazoline-6-sulphonic acid) (ABTS) assay used to analyze the ROS scavenging activity of AST and ChitoAST nanoparticles. As shown in Table 2, ChitoAST nanoparticles showed ROS scavenging activity, even though their half maximal radical scavenging concentration (RC_50_) of nanoparticles was higher than that of AST itself. The ROS scavenging activity of AST itself was slightly higher than that of L-ascorbic acid and Trolox. 

**Figure 5:**
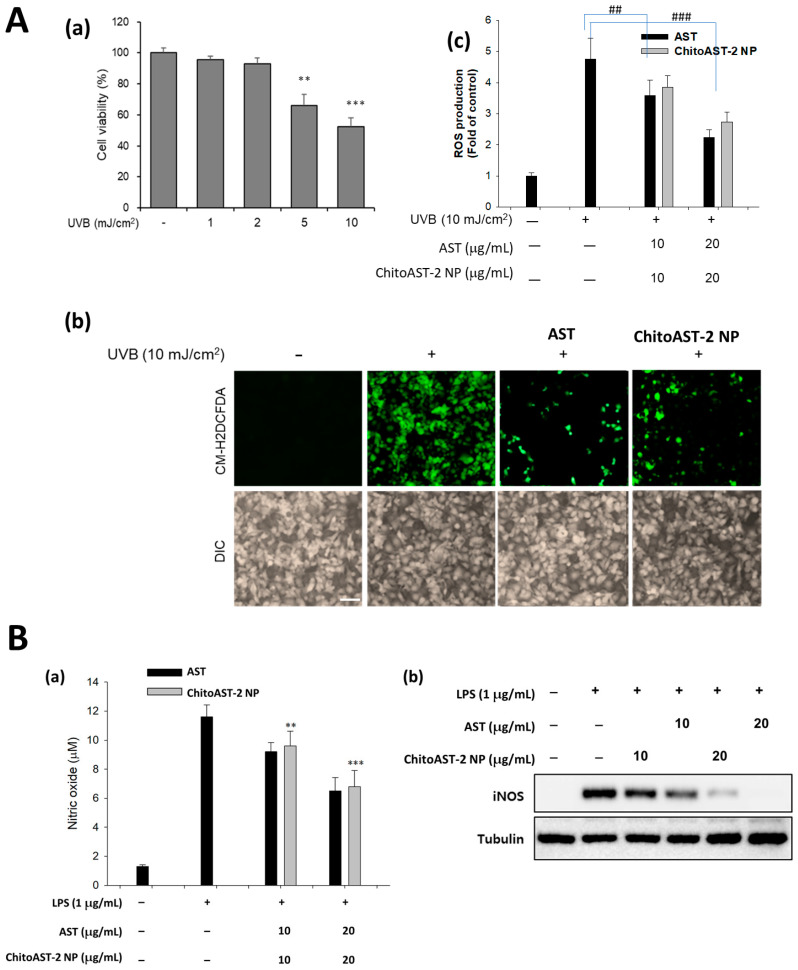
(**A**) (**Aa**) The effect of UVB irradiation against B16F10 cells. The effect of ROS scavenging effect of AST or AST released from ChitoAST nanoparticles (ChitoAST-2 NP) against UVB-irradiated B16F10 cells: (**Ab**) Fluorescence images of cells (bar = 100 µm); (**Ac**) Comparison of intracellular ROS levels. **, ***: *p* < 0.01. (**B**) (**Ba**) Inhibitory effects of AST or AST released from ChitoAST-2 nanoparticles (ChitoAST-2 NP) on nitric oxide production and (**Bb**) iNOS expression of RAW 264.7 cells. To study the effect of astaxanthin or astaxanthin released from ChitoAST-2 nanoparticles on the production of nitric oxide, cells were stimulated with LPS (1 μg/mL) for 24 h in the presence of AST or ChitoAST-2 NP. Results are expressed as percentages compared to the respective values obtained for the control. Data represent the mean ± SD over three separate experiments. ^##^, ^###^ *p* < 0.01 vs. the control group; ** *p* < 0.01, *** *p* < 0.001 vs. the LPS-treated group.

Figure 5A shows the effect of UVB irradiation on the cell viability and intracellular ROS production of the B16F10 cells. As shown in Figure 5(Aa), more than 2 mJ/cm^2^ induces cytotoxic cell death in a dose-dependent manner. These cell deaths are due to the production of intracellular ROS, as shown in Figure 5(Ab,Ac). When AST or AST released from ChitoAST-2 nanoparticles (ChitoAST-2 NP) were treated to UVB irradiated cells, intracellular ROS was significantly decreased in a dose-dependent manner, i.e., green fluorescence intensity, which represents intracellular ROS level, was decreased by the treatment of AST or ChitoAST-2 NP, as shown in Figure 5(Ab,Ac). Furthermore, intracellular ROS production also decreased dose-dependently when treated with AST or ChitoAST-2 NP. These results indicate that ChitoAST nanoparticles have as good a potential as AST itself to scavenge ROS as an antioxidant. Figure 5(Ba) shows that the scavenging activity of astaxanthin and astaxanthin released from ChitoAST-2 nanoparticles (ChitoAST-2 NP) against nitric oxide (NO) production in RAW264.7 cells was also assessed by treatment with lipopolysaccharide (LPS). As shown in Figure 5(Ba), NO production by LPS treatment on RAW264.7 cells decreases dose-dependently with treatment with ChitoAST-2 nanoparticles. These results indicate that ChitoAST-2 nanoparticles have superior antioxidant activity and inhibit reactive oxygen/nitrogen species in vitro as efficiently as AST. Figure 5(Bb) shows the iNOS expression in RAW264.7 cells. As shown in Figure 5(Bb), ChitoAST nanoparticles efficiently inhibit the expression of inducible nitric oxide synthase (iNOS) of RAW264.7 cells, as well as AST itself, indicating that ChitoAST nanoparticles have superior antioxidant activity in vitro.

### 2.3. Anticancer Activity of ChitoAST Nanoparticles

**Figure 6:**
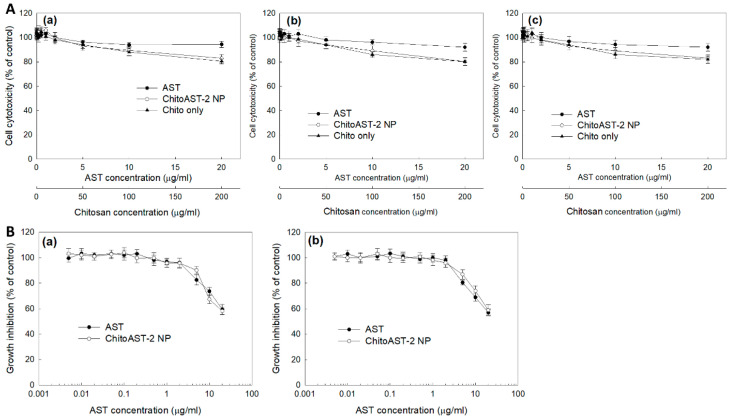
(**A**) Cytotoxicity of AST, ChitoAST nanoparticles (ChitoAST-2 NP), and empty nanoparticles (Empty NP) against (**Aa**) RAW264.7 cells, (**Ab**) B16F10 cells, and (**Ac**) HeLa cells. 2 × 10^4^ cells/well in a 96-well plate was exposed to AST, ChitoAST NP-2, and/or empty NP (Chito only) in serum-free media for 24 h. For treatment of ChitoAST NPs, ChitoAST-2 nanoparticles were used. Empty NPs are Chito only. (**B**) Growth inhibition of AST or AST released from ChitoAST-2 nanoparticles (ChitoAST-2 NP) against (**Ba**) B16F10 human melanoma cells and (**Bb**) HeLa human cervical cancer cells. A total of 4 × 10^3^ B16F10 or HeLa cells in a 96-well plate were exposed to AST or ChitoAST NP for 60 h. For treatment of ChitoAST-2 NPs, AST released from ChitoAST-2 nanoparticles (Figure 4b) in DMEM media was used for treatment of cancer cells.

Prior to analyzing anticancer activity, the effect of ChitoAST nanoparticles on the viability of normal cells and cancer cells was studied using RAW264.7 mouse macrophage cells and B16F10 human melanoma cells, as shown in Figure 6. ChitoAST nanoparticles have no significant cytotoxicity on RAW264.7 cells (Figure 6(Aa)), B16F10 cells (Figure 6(Ab)), or HeLa cells (Figure 6(Ac)); i.e., cell viability was higher than 80% on AST itself and on ChitoAST nanoparticles up to 20 µg/mL concentration. These results indicate that ChitoAST nanoparticles are not toxic until 20 µg/mL in AST concentration, just like AST itself. As shown in Figure 6B, the growth inhibition of AST released from ChitoAST-2 nanoparticles (as shown in Figure 4b) was assessed with cancer cells. Just like AST itself, AST released from ChitoAST nanoparticles dose-dependently inhibits the growth of B16F10 cells (Figure 6(Ba)) and HeLa cells (Figure 6(Bb)).

**Figure 7:**
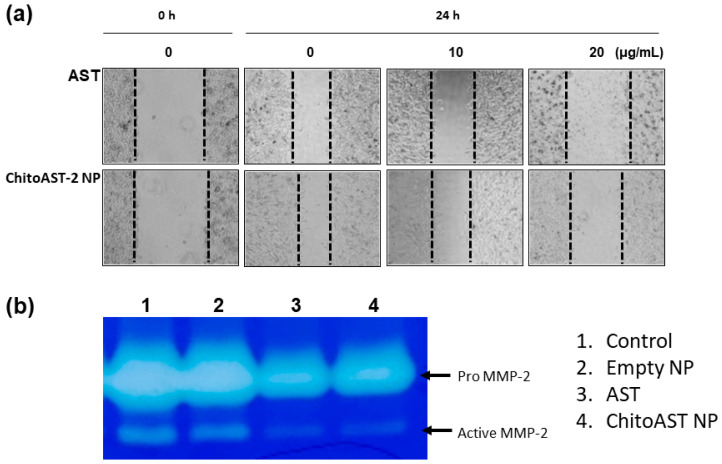
(**a**) Wound healing assay and (**b**) gelatin zymography of B16F10 cells. B16F10 cells were treated with AST or AST released from ChitoAST-2 nanoparticles (ChitoAST-2 NP). Dashed lines in wound healing assay indicated the migration of cells from 0 h to 24 h.

Figure 7 shows the inhibitory effect of AST and AST released from ChitoAST-2 nanoparticles (ChitoAST-2 NP) against the migration and matrix metalloproteinase-2 (MMP-2) activity of cancer cells. As shown in Figure 7a, AST and AST released from ChitoAST-2 nanoparticles efficiently inhibit the migration of B16F10 cells in a dose-dependent manner, even though AST only shows a higher efficacy in the inhibition of cancer cell migration. Furthermore, AST released from ChitoAST-2 nanoparticles efficiently inhibits the activity of matrix metalloproteinase-2 (MMP-2) in B16F10 cells, as shown in Figure 7b. These results indicate that ChitoAST NPs have an inhibitory effect against migration and MMP-2 expression of cancer cells in vitro.

**Figure 8:**
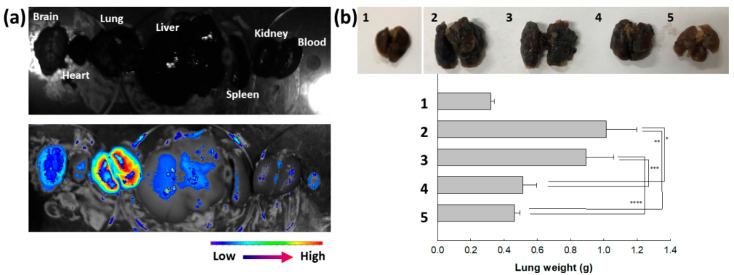
Pulmonary metastasis model of B16F10 cells for evaluation of targetability of ChitoAST-2 nanoparticles (ChitoAST-2 NPs). (**a**) Fluorescence images of each organ. (**b**) Comparison of lung weight. 1. Normal lung; 2. PBS; 3. Empty nanoparticles; 4. AST; 5. ChitoAST-2 NPs. For empty nanoparticles, Chito was intravenously (i.v.) administered. *, **, ***, ****: *p* < 0.01 For pulmonary metastasis of B16F10 cells, 5 × 10^5^ cells/0.1 mL PBS were administered intravenously (i.v.) via tail vein of nude BALb/C mice. Fluorescence dye-conjugated ChitoAST-2 nanoparticles (ChitoAST-2 NPs) were administered via tail vein of nude BALb/C mice. 1 day later, mice were sacrificed to observe fluorescence intensity of each organ, which reflects biodistribution of nanoparticles.

Their anticancer activity was also assessed with a pulmonary metastasis model of B16F10 cells, as shown in Figure 8. To study the biodistribution of ChitoAST-2 NPs, Ce6, a near-infrared (NIR) fluorescence dye, was conjugated with ChitoAST-2 NP and then intravenously administered to the mice. As shown in Figure 8a, fluorescence intensity was stronger in the lung than in other organs; i.e., ChitoAST-2 NPs were suitably concentrated in the lung and then might have targetability against cancer cells in vivo. As shown in Figure 8b, the metastasis and proliferation of B16F10 cells in the lung induce an increase in lung weight. When AST or ChitoAST-2 NP were i.v. administered to mice with pulmonary metastasis of B16F10 cells, the lung weight was significantly decreased compared to the control group. ChitoAST-2 NPs, in particular, revealed a lower lung weight than those of AST treatment, even though the gap was not significantly different. These results indicate that ChitoAST-2 NPs have appropriate anticancer and antimetastatic activity against B16F10 melanoma cancer cells. 

## 3. Discussion

The biological availability of AST is limited due to its low aqueous solubility [23,33]. β-cyclodextrin (bCD) solubilized AST 110-fold and thus increased antioxidant activity by up to 7-fold compared to free AST [33]. Zhang et al. also reported that cauliflower-like carriers (CCs) released AST in a pH-responsive manner, efficiently suppressed ROS production, and increased bioavailability [34]. ChitoAST nanoparticles can be used to solubilize AST in aqueous solution; i.e., nanoparticles can be reconstituted in deionized water up to a concentration of 1 mg AST/mL. Nanoparticles are regarded as promising vehicles to encapsulate hydrophobic agents and improve the accumulation of bioactive agents into tumors [35]. According to our results, ChitoAST nanoparticles can be formed through electrostatic interaction between amine groups of Chito and hydroxyl groups of AST, which then form spherical nanoparticles with small diameters (less than 500 nm), as shown in Figure 1. Kim et al. also reported that retinol-incorporated chitosan nanoparticles can be formed in an aqueous solution through electrostatic interaction between hydroxyl groups of retinol and amine groups of chitosan [27]. They argued that electrostatic interaction between retinol and chitosan enables the formation of small nanoparticles (less than 200 nm) and that these enhance the aqueous solubility of retinol 1600-fold. Our results also show that ChitoAST nanoparticles have a small diameter (less than 500 nm) and can reconstitute greater than 0.2 mg/mL AST concentration (as a ChitoAST-2 nanoparticle weight, 50 mg nanoparticles in 10 mL deionized water. Furthermore, they maintain specific UV absorption properties during the nanoparticle fabrication process, i.e., the UV absorption spectra of ChitoAST-2 nanoparticles in water/DMSO mixtures show similar peak characteristics to AST itself between 400 nm and 600 nm. The release rate of AST from ChitoAST nanoparticles is inversely correlated to the drug contents, i.e., the higher the drug contents, the longer the delay in the release from nanoparticles, as shown in Figure 4a. The release rate of AST from nanoparticles is practically faster in DMEM media than in phosphate-buffered saline (PBS). These results might be because DMEM media have many more salts, molecules, and fetal bovine serum (FBS), which must act as surfactants that then induce accelerated release of AST from nanoparticles. Kwak et al. also reported that cell culture media, such as RPMI1640 supplemented with FBS, accelerate the release rate of hydrophobic drugs from nanodevices [36]. Furthermore, ChitoAST nanoparticles show almost similar growth inhibition against cancer cells and superior antioxidant activity, as shown in Figure 6. 

Oxidative stress in the biological system, which is derived by loss of balance between ROS concentration and antioxidative defense, is known to cause various diseases, such as neurodegenerative disease, cardiovascular disease, and malignant disorders [37,38,39,40,41,42]. For example, oxidative stress has a deep relationship with the progress of Alzheimer’s disease and then aggravates its symptoms [37,38,39]. Oxidative stress is known to be involved in the progression of cardiovascular disease, from the initiation of atherosclerotic plague to rupture [40]. The ROS level in the biological system can be used as a biomarker to monitor the progression of cardiovascular disease [40]. Oxidative stress is also related to the initiation and progression of cancer development; i.e., elevated oxidative stress may be involved in cancer initiation by stimulating pro-oncogenic proteins [41]. Since the decreased antioxidant capacity of Parkinson’s disease patients is observed, antioxidant supplements may provide benefits to improve the status of neurodegenerative disease [42,43]. Natural products that possess antioxidants and scavenging capacity against free radicals can be used in the treatment of various liver diseases [44]. AST is also known to possess strong antioxidant activity, i.e., it neutralizes singlet oxygen, scavenges free radicals, and then affects gene expression [45,46]. Lin et al. reported that AST suppresses ROS production against oxidative stress induced by blue light and then diminishes mitochondria damage induced by exposure to blue light [47]. They argued that AST has superior productive effects, via free radical scavenging, against retinal cell damage induced by light-emitting diodes (LEDs) emitting blue light. AST is also known to have protective effects against UVB-induced oxidative stress and apoptosis in human keratinocytes [48]. Chung et al. reported that AST reduces UVB-induced ROS production and then significantly inhibits UVB-induced apoptosis of human epidermal keratinocytes [48]. Furthermore, AST has an antioxidant effect against an oxidative stress model using ARFE-19 human retinal cells [49]. Oh et al. reported that AST efficiently suppresses oxidative stress in UVB irradiation against ARFE-19 human retinal cells and then improves cell viability [49]. Our results also indicate that ChitoAST nanoparticles inhibit UVB-induced ROS generation as efficiently as AST itself and properly suppress oxidative stress in vitro (Figure 5). Even though our results were produced with B16F10 cells, ChitoAST can be applied to protect UVB-induced damage to human skin. Furthermore, AST protects mitochondria from damage induced by oxidative stress and then increases mitochondrial efficiency [49]. These strong antioxidant activities of AST are capable of improving the states of various diseases, such as malignant disorders, cardiovascular diseases, neurodegenerative diseases, traumatic brain injuries, and cancers [45,46,47,48,49,50]. Karimian et al. reported that AST induces apoptotic cell death against breast cancer cells without cytotoxicity against noncancerous cells [51]. In their reports, AST induces the expression of apoptotic proteins, such as caspase-3, caspase-9, p21, etc. Nan et al. reported that the strong antioxidant activity of AST efficiently scavenges ROS, which is excessively generated in the cochlea, and then protects patients with cisplatin-induced hearing loss (CIHL) [52]. Our results indicate that, in practice, AST itself and/or ChitoAST nanoparticles have little cytotoxicity against normal cells or cancer cells (Figure 6), while they dose-dependently inhibit the growth of cancer cells. However, they efficiently inhibit the migration of B16F10 cells, even though ChitoAST nanoparticles show decreased efficacy compared to AST itself, as shown in Figure 7. These results might be due to the sustained release properties of ChitoAST nanoparticles. Tseng et al. also reported that AST shows strong antioxidant activity and antimetastatic activity against B16F10 cells [53]. Since the MMP-2 activity of cancer cells is associated with the potential for the invasion and metastasis of cancer cells (Figure 7b), inhibition of MMP-2 activity by treatment with AST or ChitoAST nanoparticles may affect the metastasis of B16F10 cells [54]. To evaluate in vivo antimetastatic activity, ChitoAST nanoparticles were administered intravenously (i.v.) to make pulmonary metastases of B16F10 cells, as shown in Figure 8. ChitoAST nanoparticles were i.v. administered because, compared to other routes, i.v. administration of nanoparticles provides instantaneous response, wide-ranging control of drug contribution into the body, avoidance of proteolytic enzyme-mediated degradation, and rapid onset of drug action in the body [55]. As shown in Figure 8a, ChitoAST nanoparticles efficiently target lungs with B16F10 pulmonary metastasis, so these properties of ChitoAST nanoparticles must induce more efficient antimetastatic activity than AST itself.

## 4. Materials and Methods

### 4.1. Materials

Glycol chitosan (Chito, molecular weight: 250 KDa; degree of deacetylation: 88.7%) was purchased from Wako Pure Chem. Co. (Tokyo, Japan). Dialysis membrane (MWCO: 7000 g/mol), thiazolyl blue tetrazolium bromide (MTT), AST (AS), sodium dodecyl sulfate (SDS), 2,2′-azino-bis(3-ethylbenzothiazoline-6-sulphonic acid) (ABTS), and lipopolysaccharide (LPS from Escherichia coli O111:B4) were obtained from Sigma-Aldrich Co., Ltd. (St. Louis, MO, USA). All other chemicals were of analytical grade and used as received. Chlorin e6 (Ce6) was purchased from Frontier Scientific Co., Ltd. (Logan, UT, USA).

### 4.2. Preparaton of AST-Incorporated Chitosan (ChitoAST) Nanoparticles

The ChitoAST polyelectrolyte complexes were prepared according to a previous method reported by Kim et al. [27]. Briefly, Chito (100 mg) was dissolved in 10 mL of distilled water. AST (1 or 5 mg) was dissolved in 100% DMSO (1 mL) and slowly added to the Chito solution with constant stirring. After 24 h of stirring, the reaction mixtures were dialyzed for 3 days against a solution of distilled water using a cellulose dialysis tube (MWCO, 12 KDa, Sigma, St. Louis, MO, USA). The ChitoAST polyelectrolyte complex powders were obtained through lyophilization. All procedures were performed under darkened conditions.

The drug contents of AST were investigated in the following ways: ChitoAST nanoparticles (1 mg/mL) were dissolved in 5 mL of DMSO to extract AST. AST concentration was determined by measuring UV absorption at 480 nm on a microplate fluorometer (Molecular Devices, Inc., San Jose, CA, USA). Loading efficiency was calculated as follows: Drug contents (%, *w*/*w*) = (weight of incorporated AST/weight of nanoparticles) × 100.

AST release from ChitoAST nanoparticles: Nanoparticles (10 mg) were reconstituted in 5 mL of phosphate buffered saline (PBS, pH 7.4, 0.01 M) or DMEM media (supplemented with 1% penicillin/streptomycin and 10% heat-inactivated fetal bovine serum (FBS)) and introduced into a dialysis membrane (MWCO: 7000 g/mol). This was put into a 50 mL Falcon tube with 45 mL PBS or DMEM media and then agitated at 100 rpm (37 °C). Whole media was taken and then centrifuged at 15,000 rpm for 30 min. Then, CIP released from the nanoparticles was measured with a UV-spectrophotometer at 480 nm (UV-1601, Shimadzu Co., Ltd., Tokyo, Japan). For comparison, PBS or DMEM media without the drug were used similarly, as described above, and then used as a black test.

### 4.3. Preparation of Ce6-Incorporated ChitoAST Nanoparticles 

Ce6 was used as a fluorescent dye for the animal imaging study. Ce6 (2 mg) in 1 mL DMSO was added to AS3-GC nanoparticles in aqueous solution (20 mg in 10 mL water). These were sonicated (Sonics VC 505, Newtown, CT, USA) for 10 s and then magnetically stirred for 10 min. They were then dialyzed against deionized water using a dialysis membrane (MWCO: 2000 g/mol) for 1 day with exchange of water at 2–3 h intervals. Following this, the aqueous solution was adjusted to 20 mL with deionized water for evaluation of Ce6 contents in the nanoparticles. 1 mL of this solution was diluted with DMSO more than 10 times and then Ce6 concentration was measured using a fluorescence spectrophotometer (excitation wavelength: 407, emission wavelength: 664 nm) (RF-5301PC spectrofluorophotometer, Shimadzu, Kyoto, Japan). Free Ce6 was dissolved in DMSO for comparison.

Equation for Ce6 contents in the nanoparticles: Ce6 (*w*/*w*, %) = (Ce6 weight/total weight of nanoparticles)/100. Ce6 content in the nanoparticles was approximately 7.3% (*w*/*w*).

### 4.4. Characterization of Nanoparticles 

The chemical structures of Chito and AST were analyzed using Fourier transform infrared spectroscopy (FT-IR) (Shimadzu, FT-IR 8000, Japan). For measurement of FT-IR, polyelectrolyte complexes between Chito and AST were lyophilized for 3 days under dark conditions. The KBr pellets were prepared by compressing the powders under a force of 5 t in a hydraulic press. Thirty scans were obtained at a resolution of 2 cm^−1^ from 4000 to 650 cm^−1^.

The UV spectra of AST or ChitoAST nanoparticles were analyzed with a Genesys 10 s UV-VIS spectrophotometer (Thermo Fisher Scientific, Waltham, MA, USA). For the absorbance measurement of AST or ChitoAST nanoparticles in DMSO and DMSO/water mixtures, AST was dissolved in DMSO and then diluted with DMSO or deionized water because the lyophilized solid of nanoparticles was not directly dissolved in DMSO. For ChitoAST nanoparticles, ChitoAST-2 nanoparticles, prepared as described above, were diluted with deionized water or DMSO. The UV absorption spectra were scanned from 200 nm to 800 nm.

The nanoparticles were reconstituted in phosphate buffered solution (PBS, 0.01 M, pH 7.4) (1 mg/mL concentration for measurement of particle size and zeta potential. The aqueous nanoparticle solution was measured using an ELS-8000 analyzer (Photal, Osaka, Japan). The zeta potential of the ChitoAST nanoparticles was determined by Malvern zetasizer (Malvern Instruments Ltd., Malvern, UK). Each measurement was triplicated.

The morphology of AST-GC nanoparticles was observed using transmittance electron microscopy (TEM, JEM 2000 FX II, JEOL Ltd., Tokyo, Japan). The ChitoAST nanoparticle solution was mixed with a 2% (*w*/*v*) phosphotungstic acid solution and then dropped onto a copper grid coated with a carbon film. After the sample was dried at room temperature, the TEM observation was performed at 80 kV.

A field-emission scanning electron microscope (FE-SEM, S-4800; Hitachi Co., Tokyo, Japan) was used to observe the morphology of nanoparticles at 25 kV.

### 4.5. Cell Viability

RAW 264.7 mouse macrophage cells, B16F10 human melanoma carcinoma cells, and HeLa human cervical cells were obtained from the Korean Cell Line Bank (Seoul, Republic of Korea). Cells were maintained in DMEM media supplemented with 1% penicillin/streptomycin and 10% heat-inactivated fetal bovine serum (FBS) at 37 °C and 5% CO_2_.

For the cytotoxicity study, 2 × 10^4^ cells were seeded in a 96-well plate and incubated overnight at 37 °C. The culture medium was replaced with the serum-free medium containing ChitoAST nanoparticles. After 24 h of incubation, the cytotoxicity was evaluated by determining the cell viability using an MTT cell viability assay (5 mg/mL in PBS). 30 μL MTT solution was added to the well and then further cultured in 5% CO_2_ at 37 °C for 4 h. After that, supernatants were discarded, and then 100 μL DMSO was added. Cell viability was determined by measuring UV absorption at 570 nm on a microplate fluorometer (Molecular Devices, Inc., San Jose, CA, USA).

For the growth inhibition study, 3 × 10^3^ B16F10 or HeLa cells were seeded in a 96-well plate and incubated overnight at 37 °C. The culture medium was replaced with the growth media containing AST or AST released from ChitoAST-2 nanoparticles. After 48 h of incubation, the cytotoxicity was evaluated by MTT cell viability assay. For AST treatment, AST dissolved in DMSO was diluted more than 100 times (final concentration of DMSO was less than 1%, *v*/*v*). For treatment of AS released from ChitoAST-2 nanoparticles, AST concentration released from ChitoAST-2 nanoparticles in DMEM media was measured and then the concentration adjusted, similarly to the AST treatment.

### 4.6. ABTS Assay

The radical scavenging activity of the ChitoAST-2 nanoparticles was measured with an ABTS scavenging assay. ABTS (final concentration: 7 mM) and potassium persulfate (final concentration: 2.45 mM) were mixed to produce a reactive radical species for 16 h. Then, 50 μL of this solution was mixed with 50 μL ABTS solution and reacted for 5 min under dark conditions. Absorbance (ABS) was measured at 734 nm using a microplate reader (BioTek, Winooski, VT, USA) and then calculated with the equation below. Ascorbic acid and Trolox were used as positive controls.

ABTS radical scavenging activity (%) = [1 − (ABS of sample solution − ABS of blank solution)] × 100

### 4.7. UVB Irradiation Effect

UVB effect on the viability of cells: B16F10 cells (1 × 10^4^ cells/well in 96 cells) were irradiated with UVB at a dose of 0~10 mJ/cm^2^. In this experiment, the viability of cells decreased dose-dependently. When the cells were irradiated with 10 mJ/cm^2^, their viability was 52.3%, while cell viability at 1 or 2 mJ/cm^2^ and 5 mJ/cm^2^ was higher than 90% and 60%, respectively. Then, 10 mJ/cm^2^ was used for the next experiment.

Cell images for intracellular ROS level: For the observation of cell images, B16F10 cells (1 × 10^6^ cells/well in 6 wells) were treated with ChitoAST-2 nanoparticles for 4 h and then irradiated with UVB (10 mJ/cm^2^). After 1 h, cells were washed with PBS (0.01 M, pH 7.4) twice, fixed with 3.7% paraformaldehyde solution for 15 min at room temperature, and then washed with PBS again. Following this, 20 μM CM-H_2_DCFDA was added to the cells and then reacted for 30 min at 37 °C (dark condition). The cells were observed with fluorescence microscopy (Eclipse 80i; Nikon, Tokyo, Japan).

Intracellular ROS level: For measurement of intracellular ROS, B16F10 cells (1 × 10^6^ cells/well in 6 wells) were treated with ChitoAST-2 nanoparticles for 4 h and were then irradiated with UVB (10 mJ/cm^2^). After 1 h, cells were washed with PBS, 20 μM CM-H_2_DCFDA added and then reacted for 30 min at 37 °C (Dark condition). Following this, the cells were washed with PBS, harvested by trypsinization, and then measured with a spectrofluorophotometer (Excitation wavelength: 485 nm, emission wavelength: 535 nm). The intracellular ROS level was expressed as relative fluorescence intensity compared to the control treatment.

### 4.8. Nitric Oxide (NO) Assay

The anti-inflammatory activity of ChitoAST-2 nanoparticles was evaluated using NO assay. RAW264.7 cells (1 × 10^6^ cells/mL) were seeded in 24-well plates and incubated at 37 °C (5% CO_2_) for 18 h. Cells were exposed to ChitoAST-2 nanoparticles (0, 25, 50, 100, 200 ppm) in serum-free media for 3 h and then treated with LPS (1.0 μg/mL) for 24 h. Supernatants (100 μL) were mixed with 100 μL of Griess reagent [1% (*w*/*v*) sulfanilamide, 0.1% (*w*/*v*) naphylethylenediamine in 2.5% (*v*/*v*) phosphoric acid] and then reacted for 10 min under dark conditions. This was measured with a microplate reader (BioTek, Winooski, VT, USA) at 540 nm.

### 4.9. Wound Healing Assay and Gelatin Zymography

A wound healing assay was performed to evaluate the effect of ChitoAST-2 nanoparticles on the migration potential of B16F10 cells using ibidi Culture Inserts (ibidi GmbH, Planegg/Martinsried, Germany). Ibidi Culture Inserts were placed in 6 wells, and then 5 × 10^4^ B16F10 cells were seeded in each side of the ibidi Culture Inserts. This was incubated at 37 °C and 5% CO_2_ overnight. After that, AST or ChitoAST-2 nanoparticles in serum-free media were treated to cells and incubated at 37 °C and 5% CO_2_ for 24 h. The dose of AST was 20 µg/mL in the media. Following this, cells were carefully washed with PBS, and then the field of wound healing and cell migration was observed using light microscopy (Olympus CKX 53, Olympus, Tokyo, Japan).

A gelatin zymography was performed to evaluate the effect of ChitoAST-2 nanoparticles on the invasive and metastatic potentials of B16F10 cells as follows: A total of 1 × 10^6^ B16F10 cells in 6-well plates were treated with AST or ChitoAST-2 nanoparticles in serum-free media for 24 h. The dose of AST was 20 µg/mL in the media. The media were harvested to measure MMP activity and developed with substrate gel electrophoresis using SDSPAGE containing 10% gelatin. The protein concentration of the conditioned media was measured with a Bicinchoninic Acid (BCA) protein assay kit (Sigma-Aldrich Chem. Co., Ltd. (St. Louis, MO, USA). Protein concentration in conditioned media was adjusted to the same concentration and then mixed with Laemmli buffer (Bio-Rad Lab. Co., Hercules, CA, USA). These were loaded onto the gel and then separated by electrophoresis. Following this, the gels were soaked three times for 30 min in Triton buffer (2.5% Triton X-100 in PBS) to remove SDS and then incubated for 24 h at 37 °C. These were stained with 0.1% Coomassie Brilliant Blue R-250, and destained bands were obtained as clear bands.

### 4.10. Animal Pulmonary Metastasis Model and In Vivo Animal Tumor Imaging

The pulmonary metastasis model using nude BALb/C mice (male, 20 g, 5-week-old) was prepared using B16F10 cells for in vivo fluorescence imaging. B16F10 cells (5 × 10^5^ cells/0.1 mL PBS) were intravenously (i.v.) administered via tail vein of nude BALb/C mice. 3 weeks later, fluorescence dye-conjugated ChitoAST-2 nanoparticles (10 mg/kg) were sterilized with a 1.2 µm syringe filter and i.v. administered via tail vein of nude BALb/C mice. Injection volume was 0.1 mL. After 1 day, mice were sacrificed and dissected to observe the organs and the biodistribution of nanoparticles with a Maestro^TM^ 2 small animal imaging instrument (Cambridge Research & Instrumentation, Woburn, MA, USA).

For comparison of lung weight, B16F10 cells (5 × 10^5^ cells/0.1 mL PBS) were intravenously (i.v.) administered via tail vein of nude BALb/C mice. Five mice were used for each group. AST dissolved in a 5% HCO-60 (Nicco Chem. Co., Tokyo, Japan) solution was diluted with PBS (0.01 M, pH 7.4). ChitoAST-2 nanoparticles were sterilized with a 1.2 µm syringe filter. Five days after cancer cell administration, this solution was injected into the mice (AST dose: 10 mg/kg). PBS was injected for comparison as a control. The injection volume was 0.2 mL. After 4 weeks, the mice were sacrificed to separate their organs. Lung weights were measured for the analysis of pulmonary metastasis of B16F10 cells.

## 5. Conclusions

ChitoAST nanoparticles were fabricated to solve the aqueous solubility of AST and improve its biological activity. Nanoparticles were formed by polyelectrolyte complex formation between Chito and AST and showed small diameters (less than 400 nm). ChitoAST nanoparticles had no significant cytotoxicity against RAW264.7 cells, B16F10 melanoma cells, and HeLa human cervical cells, while ChitoAST nanoparticles dose-dependently inhibited cancer cell proliferation. ChitoAST nanoparticles properly inhibited intracellular ROS production, nitric oxide (NO), and iNOS in vitro. ChitoAST nanoparticles inhibited the migration of cancer cells in a wound healing assay. In the animal pulmonary metastasis model, ChitoAST nanoparticles were efficiently delivered to the lung and then efficiently inhibited pulmonary metastasis. We suggest that ChitoAST nanoparticles are promising candidates for antioxidative and anticancer therapy of B16F10 cells.

## Figures and Tables

**Table 1:** Characterization of ChitoAST nanoparticles.

Formulation	AST/GC (mg/mg)	Particle Size (nm)	Drug Contents ^a^ (%, *w*/*w*)	Loading Efficiency ^b^ (%, *w*/*w*)
Empty NP ^c^	0/100	-	-	-
ChitoAST-1	1/100	380 ± 35	0.92	92.0
ChitoAST-2	5/100	270 ± 23	4.3	86.4

^a^ Drug contents = (DC, % (*w*/*w*)) = (AST weight in nanoparticles/Total weight of nanoparticles) × 100. ^b^ Loading efficiency (LE, % (*w*/*w*)) = (AST weight in nanoparticles/feeding weight of AST) × 100. ^c^ Empty NP = empty nanoparticles. The empty NP was prepared similarly to Chito itself.

**Table 2:** ABTS assay.

Sample	ABTS RC_50_ (µg/mL) ^a^
AST	11.8
ChitoAST-2 NP	29.3
L-ascorbic acid	4.1
Trolox	8.4

^a^ ABTS RC_50_ (µg/mL) = half maximal radical scavenging concentration of AST or AST-GC 3 nanoparticles.

## Data Availability

Data are contained within the article.
